# Genes and signaling networks regulated during zebrafish optic vesicle morphogenesis

**DOI:** 10.1186/1471-2164-15-825

**Published:** 2014-09-30

**Authors:** Jun Yin, Maria E Morrissey, Lisa Shine, Ciarán Kennedy, Desmond G Higgins, Breandán N Kennedy

**Affiliations:** UCD Conway Institute, UCD School of Medicine and Medical Science, University College Dublin, Belfield, Dublin 4 Ireland; UCD Conway Institute, UCD School of Biomolecular and Biomedical Science, University College Dublin, Belfield, Dublin 4 Ireland; Department of Genetics, Yale University School of Medicine, New Haven, CT 06520 USA

## Abstract

**Background:**

The genetic cascades underpinning vertebrate early eye morphogenesis are poorly understood. One gene family essential for eye morphogenesis encodes the retinal homeobox (Rx) transcription factors. Mutations in the human retinal homeobox gene (RAX) can lead to gross morphological phenotypes ranging from microphthalmia to anophthalmia. Zebrafish *rx3* null mutants produce a similar striking eyeless phenotype with an associated expanded forebrain. Thus, we used zebrafish *rx3*^-/-^ mutants as a model to uncover an Rx3-regulated gene network during early eye morphogenesis.

**Results:**

Rx3-regulated genes were identified using whole transcriptomic sequencing (RNA-seq) of *rx3*^-/-^ mutants and morphologically wild-type siblings during optic vesicle morphogenesis. A gene co-expression network was then constructed for the Rx3-regulated genes, identifying gene cross-talk during early eye development. Genes highly connected in the network are hub genes, which tend to exhibit higher expression changes between *rx3*^-/-^ mutants and normal phenotype siblings. Hub genes down-regulated in *rx3*^-/-^ mutants encompass homeodomain transcription factors and mediators of retinoid-signaling, both associated with eye development and known human eye disorders. In contrast, genes up-regulated in *rx3*^-/-^ mutants are centered on Wnt signaling pathways, associated with brain development and disorders. The temporal expression pattern of Rx3-regulated genes was further profiled during early development from maternal stage until visual function is fully mature. Rx3-regulated genes exhibited synchronized expression patterns, and a transition of gene expression during the early segmentation stage when Rx3 was highly expressed. Furthermore, most of these deregulated genes are enriched with multiple RAX-binding motif sequences on the gene promoter.

**Conclusions:**

Here, we assembled a comprehensive model of Rx3-regulated genes during early eye morphogenesis. Rx3 promotes optic vesicle morphogenesis and represses brain development through a highly correlated and modulated network, exhibiting repression of genes mediating Wnt signaling and concomitant enhanced expression of homeodomain transcription factors and retinoid-signaling genes.

**Electronic supplementary material:**

The online version of this article (doi:10.1186/1471-2164-15-825) contains supplementary material, which is available to authorized users.

## Background

In vertebrates, eyes form as forebrain evaginations that produce optic vesicles. Upon contacting the surface ectoderm, the optic vesicles form the neural retina and retinal pigment epithelium [1]. The retinal homeobox transcription factor, RX (also known as retina and anterior neural fold homeobox, RAX) has an evolutionarily conserved role during early vertebrate eye development [2]. Of clinical relevance, mutations in human RX can cause anophthalmia (a complete absence of eyes) or microphthalmia (a small eye) [3, 4]. Homozygous murine Rx nulls fail to form eye structures, have severe brain defects, including the absence of forebrain and midbrain structures, and die neonatally [2]. In contrast, hypomorphs are viable, but lack eyes and optic tracts [5].

Teleost genomes can contain multiple *Rx* gene paralogs with specialized roles [6]. In, medaka and zebrafish, *rx3* mutants lack eyes because of a failure of optic vesicles to properly form [7, 8]. Zebrafish *rx1* and *rx2* exhibit reduced expression in *rx3* mutants indicating that Rx3 is an upstream regulator of these paralogs [9]. When expression of *rx1* and *rx2* are knocked-down in zebrafish, optic vesicles do form, but microphthalmia results [6]. Knocking down the expression of *rx1* and *rx2* at later stages of eye development confirms that *rx1* and *rx2* have diversified, pleiotropic roles: *rx1* has a distinct function in the proliferation of retinal progenitor cells [10]. In zebrafish *rx3* null mutants, in addition to defective optic vesicle morphogenesis, the forebrain is enlarged [11]. This is consistent with Rx3 functioning to segregate the eye field from the telencephalon. Indeed, Rx3 alters the migratory behaviour of retinal progenitor cells towards optic vesicle evagination and away from the default forebrain fate [7, 11]. However, the Rx3-regulated genetic networks driving optic vesicle evagination are still poorly understood.

Rx functions as part of a highly conserved network of transcription factors, collectively referred to as the eye field transcription factors, and includes Pax6, Six3, Optx2, Tlx, Lhx2, and ET [12]. A small number of zebrafish Rx3 targets have previously been identified by comparing candidate gene expression in *rx3*^*-/-*^ mutants and wild-type phenotype siblings around somitogenesis. Zebrafish Rx3 functions to up-regulate genes promoting eye development, and concomitantly down-regulate genes supporting forebrain development [11]. *cxcr4a is* down-regulated at about 10 hours post fertilization (hpf), *mab21l2 and pard6gb* are down-regulated at 11 hpf, followed by *rx2* at 13 hpf and *rx1* at 14–15 hpf [9, 13–15]. In *rx3* mutants, *vsx2* expression is not detectable in the zebrafish optic cup at 26 hpf, though in medaka *rx3* mutants, *vsx2* expression has been shown to be down-regulated at a much earlier stage of development (16-somites) [9, 16]. Knockdown of *rx1, rx2, mab21l2* or *vsx2* genes produces small eye phenotypes in zebrafish supporting their role in promoting eye development [6, 13, 17]. Markers of forebrain development, including *emx3, foxg1, tlc, epha4a, ephb4a* and *nlcam* are up-regulated in *rx3*^*-/-*^ mutants during very early stages of eye morphogenesis from 10–12 hpf onwards [8, 11, 18]. The onset of Rx3 expression is from ~9 hpf, during the late gastrulation stage [19]. However, quantitative comparisons of the temporal expression of Rx3 and reported targets during eye developmental stages have not been performed. Furthermore, the Rx3 target genes that have been reported are likely to represent a small proportion of all the genes regulated by Rx3 during early eye development.

RNA-seq enables quantitative, whole transcriptome level measurement of known and novel gene expression [20]. Here, we applied RNA-seq to gain a more comprehensive understanding of the genes regulated by Rx3 in zebrafish. Comparing recessive *rx3*^*-/-*^ mutants and morphologically wild-type siblings, we identified genes with significant differential expression during early eye morphogenesis. We profiled the temporal expression of *rx3* and the Rx3-regulated genes using RNA-seq data sets covering maternal to post-segmentation development stages. Finally, we assembled a gene co-expression network that highlights the complex positive and negative interaction of Rx3-regulated genes with homeodomain transcription factors, retinoid and Wnt signaling factors.

## Results

### Whole transcriptome sequencing of 13 hpf Zebrafish *rx3*^*-/-*^mutants

**Figure 1:**
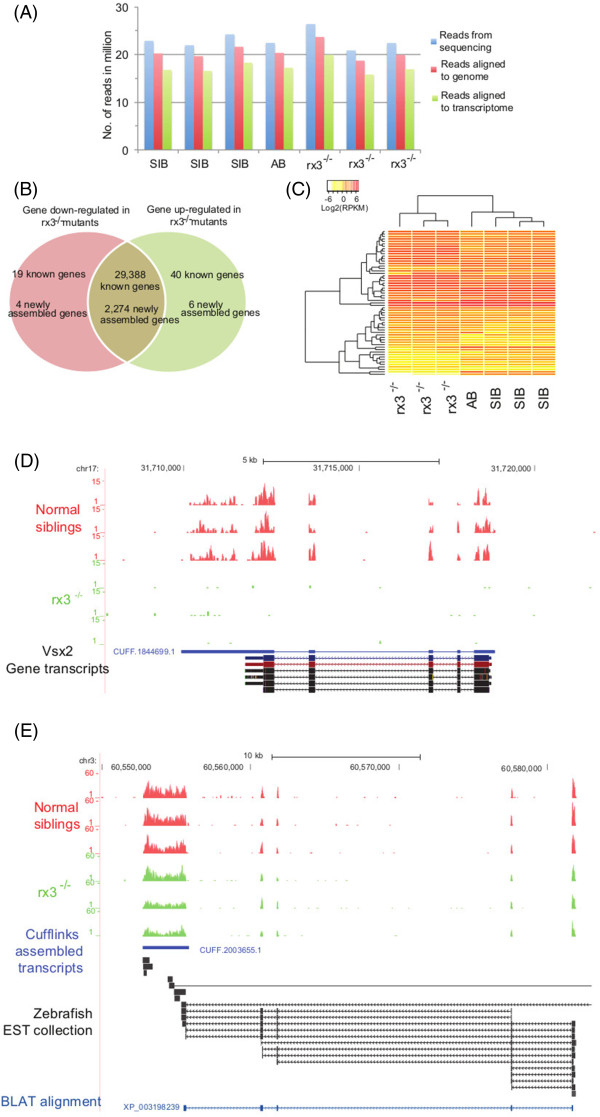
**Successful whole transcriptome sequencing of** ***rx3*** ^**-/-**^ **optic vesicle morphogenesis mutants. (A)** Summary of sequencing reads alignment for wild-type phenotype siblings (SIB), wild-type AB strain (AB) and *rx3* ^-/-^. **(B)** Venn diagram showing the number of differentially expressed genes between *rx3* ^*-/-*^ mutants and siblings. **(C)** Hierarchical clustering of the RNA-seq gene expression data reveals gene cohorts that are deregulated in *rx3* mutants, and that have similar expression patterns in siblings and AB larvae. Hierarchical clustering was based on Euclidean distance with complete linkage. **(D)** Representative RNA-seq read alignments from normal phenotype siblings and *rx3* ^*-/-*^ mutants are depicted in red and green respectively. Transcripts assembled by Cufflinks are in blue. *vsx2* is down-regulated in *rx3* ^*-/-*^ mutants versus siblings. **(E)** A representative profile of the RNA-seq reads for a novel assembled transcript shown to be down-regulated in *rx3* ^*-/-*^ mutants (green traces) versus siblings (red traces). This novel gene is zebrafish hypothetical protein XP_003198239 identified from BLAT alignments and by the zebrafish un-spliced EST collection within the UCSC genome browser.

To identify genes regulated by Rx3 during optic vesicle morphogenesis, adult zebrafish carriers of a null *rx3* mutation were mated. Offspring were phenotypically separated into pools consisting of mutants with an absence of optic vesicles or siblings exhibiting a wild-type phenotype at 13 hours post fertilization (hpf), the earliest time point at which optic vesicle evagination phenotypes can be reliably and quickly detected to ensure correct sampling at this timepoint. Three replicates of pooled RNA samples from 13 hpf eyeless mutants (*rx3*^-/-^) or phenotypically wild-type siblings (*rx3*^*+/+*^*or rx3*^*+/-*^ in unknown ratios), and one replicate of 13 hpf wild-type zebrafish larva were collected for whole transcriptome sequencing. These samples were sequenced to a depth of ~21-26 million reads (Figure 1A). About 19–24 million reads could be aligned to the zebrafish genome Zv9, representing 89-91% of all generated reads (Figure 1A).

Ample genome annotation is essential for interpreting large gene expression datasets [21]. Thus, we constructed a more comprehensively annotated zebrafish transcriptome by combining *de novo* transcripts assembled using Cufflinks with known transcripts from public databases RefSeq, Ensembl and GenBank. The transcribed regions in the zebrafish genome were increased from only 56 Mb as covered by Ensembl transcripts to 76 Mb after integrating all the transcripts. A total of 31,731 genes were defined using the combined set of transcripts. Of these, 2284 genes were solely assembled using RNA-seq reads. Consequently, ~16-20 million reads were mapped to the customized transcriptome representing 82-84% of the aligned reads (Figure 1A). Overall, ~3-4% of reads were mapped to novel transcribed regions, which are either extensions of known genes forming novel exons and/or longer UTRs (Figure 1D), or entirely novel genes assembled from the RNA-seq data (Figure 1E).

### Identification of differentially expressed genes in *rx3*^*-/-*^optic vesicle mutants

Next, we sought to identify genes significantly deregulated during optic vesicle development due to the absence of functional Rx3. To measure gene expression levels, read counts for each gene were normalized using reads per kilobase per million reads (RPKM) (Figure 1B-E). Genes with extremely low read counts were eliminated from further analysis due to a lack of reproducibility (Additional file 1: Figure S1). Using an RPKM cut-off of 5, ~28% of the genes are highly expressed, whereas, with a moderately stringent cut-off of 0.5 ≤ RPKM <5, ~52% of the annotated genes are expressed. Generally, *rx3*^*-/-*^ mutants and wild-type phenotype siblings have no significant differences in global gene expression levels (Additional file 1: Figure S1).

We compared individual gene expression levels in *rx3*^*-/-*^ mutants and wild-type phenotype siblings using the Bioconductor package DEGseq [22]. In summary, 69 genes were significantly differentially expressed between *rx3*^*-/-*^ mutants and the morphologically normal siblings, with 23 genes significantly down-regulated and 46 significantly up-regulated with ≥1.5 fold change (Figure 1B, Table 1 and Additional file 1: Figure S1). These differentially expressed genes exhibit similar expression profiles in both 13 hpf wild-type larvae and wild-type phenotype siblings compared to *rx3*^*-/-*^ mutants (Figure 1C). Table 1 summarizes the expression levels of the 20 most significant differentially expressed genes. The annotation resources for the newly assembled genes are very limited (Additional file 2: Table S1). Thus, in subsequent analyses we focused on the 19 down-regulated and 40 up-regulated genes with open-access annotated transcripts (Figure 1B-C).

**Table 1:** **Top 20 differentially expressed genes between normal phenotype siblings and** ***rx3*** ^***-/-***^ **mutants selected by Q-value**

Gene Symbol	Description	Chromosome location	RPKM of normal siblings	RPKM of r***x3*** ^***-/-***^	Log2 (FC)	Q-value	Adhesion	Death	Development	Metabolism	Regulation	Response to stress/stimulus	Signal transduction	Transcription	Transport	Vision and light stimulus
*rx2*	Retinal homeobox gene 2	chr2:56043918-56074494	4.76	0.02	8.01	2.57E-264			+	+	+					
*vsx2*	Visual system homeobox 2	chr17:31709934-31718872	2.90	0.11	4.72	2.81E-122			+		+	+				+
*hmx1*	H6 homeo box 1	chr1:41290725-41295809	1.91	0.37	2.36	4.45E-55			+		+					
*ccdc90b*	Coiled-coil domain containing 90B	chr21:22003868-22007106	4.96	1.43	1.80	7.73E-32										
*rorab*	RAR-related orphan receptor A, paralog b	chr7:30875138-30908102	5.26	1.78	1.56	1.06E-82				+	+	+	+	+		
*bcr*	Breakpoint cluster region	chr8:31348750-31443924	6.22	3.00	1.05	4.46E-90				+	+	+	+			
*mab21l2*	Mab-21-like 2	chr1:22850233-22852357	19.26	9.88	0.96	9.99E-72			+							
*six7*	Sine oculis homeobox homolog 7	chr7:8229741-8248372	9.84	5.22	0.92	8.85E-65			+		+					
*traf4a*	Tnf receptor-associated factor 4a	chr15:14535600-14612424	13.88	8.15	0.77	1.22E-53				+	+	+	+			
*pou3f3a*	POU class 3 homeobox 3a	chr9:6696999-6702825	3.86	5.89	-0.61	6.89E-32			+	+	+					
*dmrta2*	DMRT-like family A2	chr8:16775899-16778945	10.97	18.97	-0.79	4.71E-62			+		+					
*fezf2*	FEZ family zinc finger 2	chr11:20477076-20479907	10.53	18.38	-0.80	8.49E-73			+	+	+	+				
*crtac1*	Cartilage acidic protein 1	chr1:55473931-55487036	61.86	108.31	-0.81	0.00E + 00										
*wnt7ba*	*Wingless-type MMTV integration site family, member 7Ba*	chr4:17770167-17784191	4.56	8.34	-0.87	6.57E-39			+	+	+	+	+			
*ntn1b*	netrin 1b	chr3:25810033-25915079	3.95	7.37	-0.90	3.47E-62			+							
*sox1b*	SRY-box containing gene 1b	chr1:46690904-46693589	7.71	16.24	-1.07	8.19E-82			+	+	+					
*foxg1a*	Forkhead box G1a	chr17:29175718-29178733	7.43	17.41	-1.23	8.14E-139			+	+	+	+		+		
*fezf1*	FEZ family zinc finger 1	chr25:29139552-29143219	5.09	12.80	-1.33	5.51E-97			+	+	+					
*zgc:165461*	zgc:165461	chr17:23128496-23132150	4.44	11.97	-1.43	9.20E-71										
*nr2e1*	Nuclear receptor subfamily 2, group E, member 1	chr20:32476129-32485397	4.71	13.33	-1.50	4.42E-142				+	+	+	+			

Several homeodomain transcription factors (*e.g. rx2, vsx2* and *hmx1*) are within the most prominent down-regulated genes in *rx3*^*-/-*^ mutants (Table 1). The retinoic acid receptor-related orphan receptors, *rorab* and *rorb*, also show lower expression in *rx3*^*-/-*^ mutants (Table 1). Importantly the list of the most deregulated genes (Table 1) includes several known Rx3 targets (*rx2, mab21l2* and *foxg1a*)*,* validating the RNA-seq results [11, 13]. Most other differentially expressed genes are potential novel targets regulated by Rx3, directly or indirectly. Moreover, several down-regulated homeobox genes have predominant expression in the eye during optic vesicle morphogenesis consistent with an important role during early eye development (*rx2-3* [19], *six6* [23], *hmx1* [24], *six7* [25], *vsx2* [26]).

In order to investigate the association of the deregulated genes with genetic diseases, we identified human homologs of the zebrafish genes, and obtained genetic disease information from public databases (Table 2). Genes down-regulated in *rx3*^*-/-*^ mutants are mostly associated with eye diseases, including microphthalmia (*rx2*, *rx3*, *vsx2*, *six6b* and *aldh1a3*) and oculoauricular syndrome (*hmx1*). RX mutations are a major cause of microphthalmia in humans [3, 4]. The association of microphthalmia with Rx3-regulated genes underlines the important role of the Rx3 signaling network in severe inherited ocular disease. Further understanding of Rx3 and Rx3-regulated genes can provide insights into the molecular pathogenesis of these diseases.

**Table 2:** **Differentially expressed genes associated with human diseases**

Gene	Description	Log2 (FC)	Qvalue	OMIM	Title
*rx2*	Retinal homeobox gene 2	8.01	2.57E-264	611038	Microphthalmia, isolated 3 (MCOP3)
*vsx2*	Visual system homeobox 2	4.72	2.81E-122	610092	Microphthalmia, isolated, with coloboma 3 (MCOPCB3)
				610093	Microphthalmia, isolated 2 (MCOP2)
*hmx1*	H6 homeo box 1	2.36	4.45E-55	612109	Oculoauricular syndrome
*aldh1a3*	Aldehyde dehydrogenase 1 family, member A3	2.33	1.23E-22	610093	Microphthalmia, isolated 8 (MCOP8)
*six6b*	Sine oculis-related homeobox 6b	1.70	2.72E-04	212550	Microphthalmia, isolated, with cataract 2 (MCOPCT2)
*rx3*	Retinal homeobox gene 3	0.65	1.08E-18	611038	Microphthalmia, isolated 3 (MCOP3)
*arx*	Aristaless related homeobox	-0.88	8.28E-28	300004	Corpus callosum, agenesis of, with abnormal genitalia
				300215	Lissencephaly, x-linked, 2 (LISX2)
				300419	Mental retardation, x-linked, with or without seizures, ARX-related
				308350	Epileptic encephalopathy, early infantile, 1 (EIEE1)
				309510	Partington x-linked mental retardation syndrome (PRTS)
*foxg1a*	Forkhead box G1a	-1.23	8.14E-139	613454	Rett syndrome, congenital variant

### Molecular validation of genes down-regulated in 13 hpf *rx3*^*-/-*^mutants

**Figure 2:**
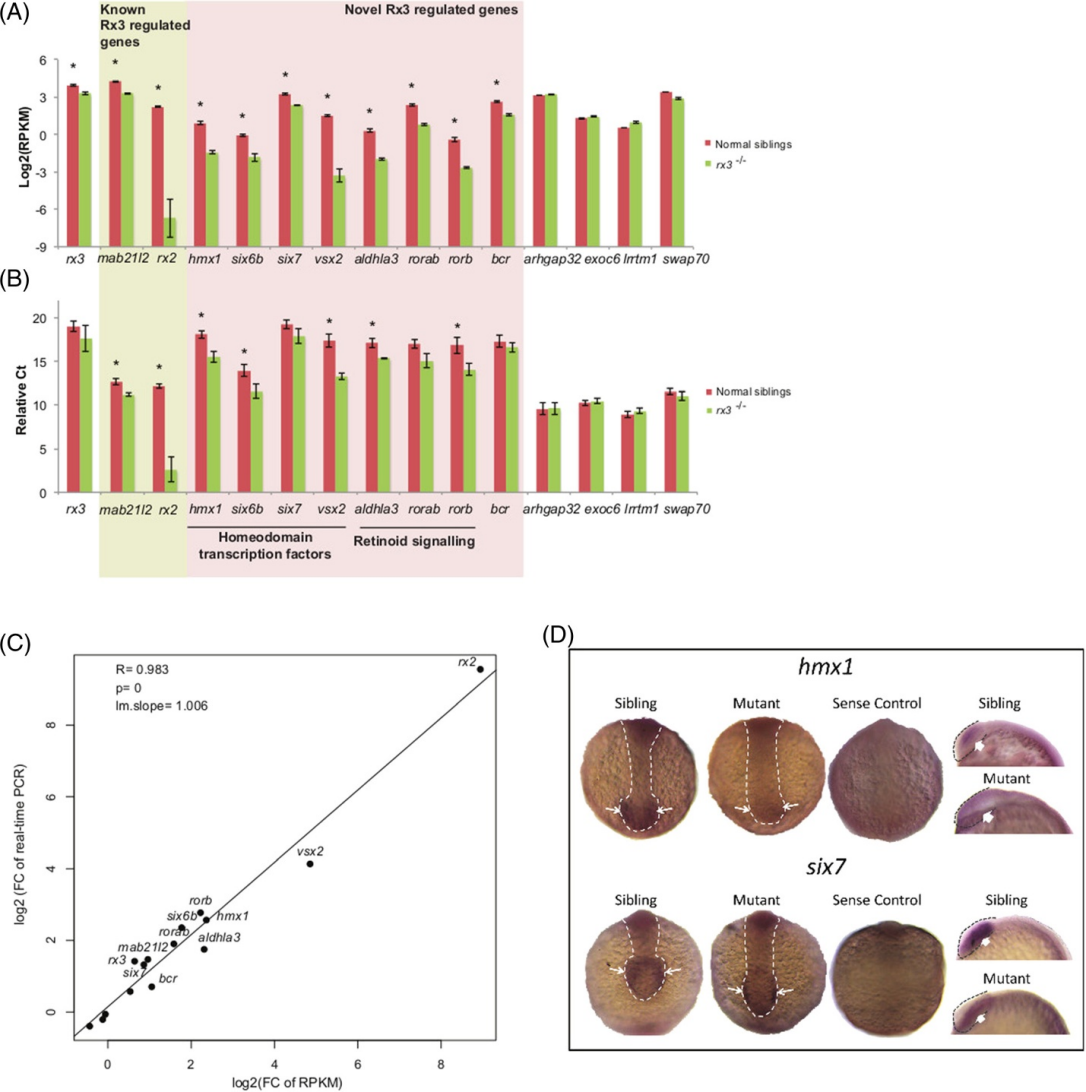
**Molecular validation of the RNA-seq data. (A-B)** Bar graphs of the relative expression levels of selected genes at 13 hpf in *rx3* ^-/-^ and siblings. **(A)** Relative expression levels of the genes from the RNA-seq plotted as log2 transformed RPKM. *, q-value < 0.001 by DEGseq. **(B)** Relative expression levels of the same genes quantified by real-time PCR and plotted as Ct values relative to the least abundant gene. *, Student’s t test p-value < 0.05. **(C)** Dot-plot showing the correlation between the expression levels determined from the RNA-seq and real-time PCR analyses. **(D)** *In situ* hybridisation confirms the down-regulation of the homeodomain genes *six7* and *hmx1* at the 6–8 somite stage using DIG-labelled anti-sense probes to these genes. In the eye field *hmx1* transcription show normal expression in wild-type phenotype siblings but are prominently down-regulated in *rx3* ^-/-^ mutants. Down-regulation of *six7* was also evident in the eye field, where siblings show normal expression and mutants show some residual expression in the ventral eye. Sense negative controls showing the expression patterns obtained with sense probes are also shown. Arrows indicate the eye field, hollow arrows for dorsal images and full arrows for lateral images.

Due to an interest in eye development, we focused on validating the significant down-regulation of selected genes in 13 hpf *rx3*^*-/-*^ mutants, which are mostly involved in visual function and eye development. Initially, quantitative RT-PCR was conducted on three independent RNA replicates from *rx3*^*-/-*^ mutants and wild-type phenotype siblings. Two of the highest ranking genes, *rx2* and *mab21l2,* are known by wholemount *in situ* hybridisation to have reduced expression in *rx3*^*-/-*^ mutants and acted as positive controls [13]. In addition to *rx2* and *mab21l2*, other homeodomain transcription factors (*e.g. hmx1, rx3, six6b, six7* and *vsx2*), retinoid-signaling factors (*e.g. aldh1a3, rorab* and *rorb*) and another down-regulated gene (*bcr*) were chosen. Several non-differentially expressed genes with predominant optic primordium expression (*e.g. arhgap32*, *exoc6*, *lrrtm1* and *swap70*), were selected as negative controls [27, 28]. Notably, significant down-regulation of *mab21l2, rx2*, *hmx1, six6b, vsx2, aldh1a3* and *rorb* transcripts in 13 hpf *rx3*^*-/-*^ mutants was confirmed by real-time PCR (Figure 2A). The results from real-time PCR and RNA-seq were consistent, with very similar fold changes observed (Figure 2A-C). Indeed, the correlation of the fold changes from these two methods was 0.98 highlighting the reproducibility of the RNA-seq data (Figure 2B). The RNA-seq analyses produced more significant differences due to the reduced data variation and specialized statistical analyses used with this data type.

Subsequently, we selected *hmx1* and *six7* for further analysis by *in situ* hybridisation. This confirmed the down-regulation of *hmx1 and six7* expression during early eye development in *rx3*^*-/-*^ mutants (Figure 2D). The residual *six7* expression in the apparent vestigial eye field located on the ventral side of the anterior head likely explains why PCR was not sufficiently sensitive to detect the significant *six7* deregulation observed by RNA-seq and *in situ* hybridisation (Figure 2A, B, D). Overall, most genes demonstrated to be down-regulated in *rx3*^*-/-*^ mutants by bioinformatic analysis of RNA-seq data have been validated by independent molecular techniques.

### Gene set analysis of differentially expressed genes

**Figure 3:**
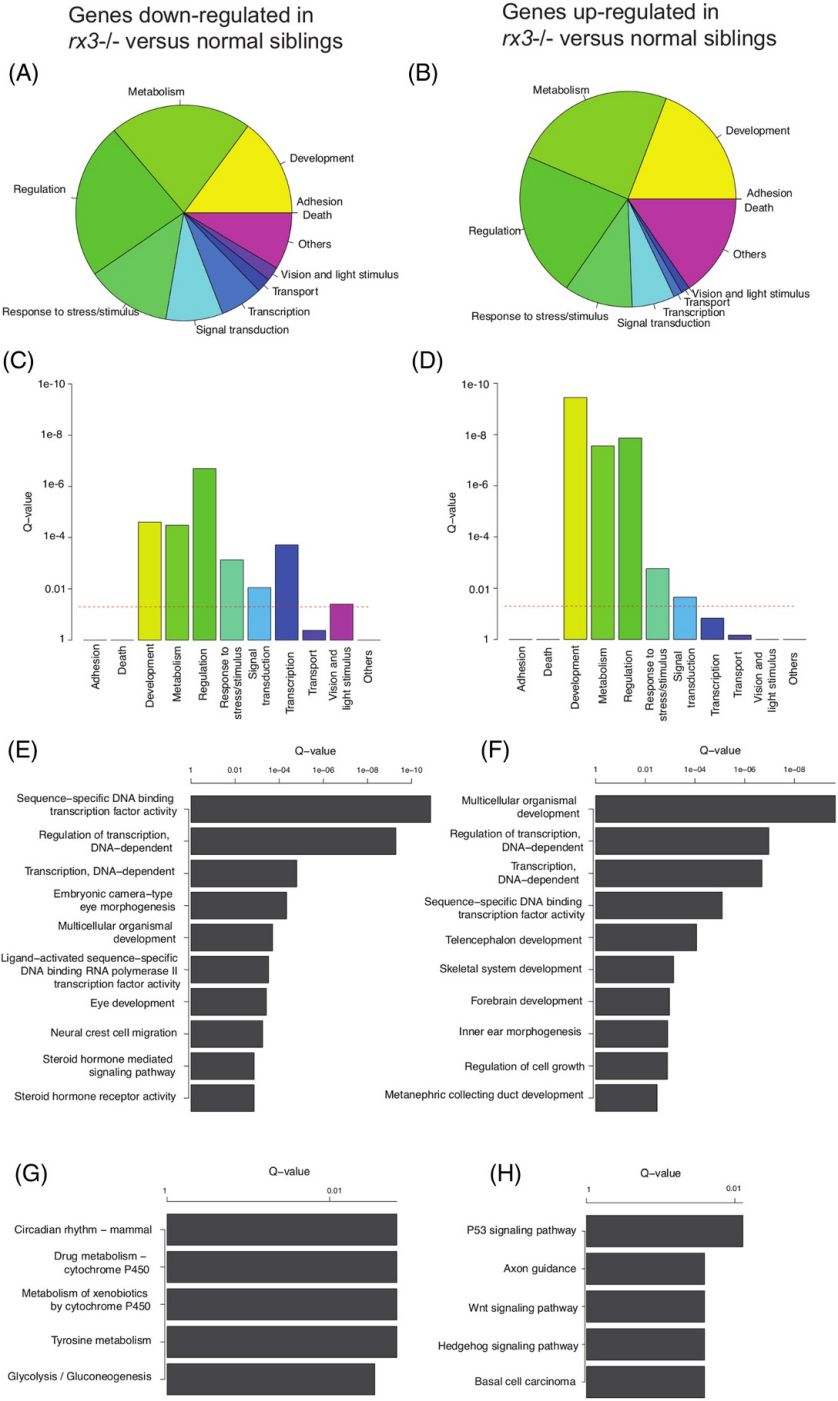
**Biological process and pathway summary of the genes differentially expressed between siblings and** ***rx3*** ^***-/-***^ **mutants.** Left column: genes down-regulated in *rx3* ^*-/-*^ mutants versus siblings. Right column: genes up-regulated in *rx3* ^*-/-*^ mutants versus siblings. **(A)** and **(B)** are pie charts representing the percentage of the differentially expressed genes in selected GO biological process categories. **(C)** and **(D)** are Q-value bar graphs for the selected GO biological process categories using a Fisher’s exact test. **(E)** and **(F)** are bar graphs of the Fisher’s exact Q-values for the top GO biological process terms enriched in the significantly deregulated genes. **(G)** and **(H)** are bar graphs of the Fisher’s exact Q-values for the top KEGG pathways enriched in the *rx3* ^-/-^ deregulated genes.

Genes which are deregulated in *rx3*^*-/-*^ mutants provide a novel resource to identify biological processes underpinning optic vesicle morphogenesis. Thus, the Gene Ontology (GO) database was queried with those genes differentially expressed in 13 hpf *rx3*^*-/-*^ mutants (Figure 3A-F). Generally, genes down-regulated and up-regulated in *rx3*^*-/-*^ mutants are both enriched in the biological processes “*Metabolism*”, “*Regulation*” and “*Development*” (Figure 3A-D). However, *“Vision and Light Stimulus”* and *“Transcription”* are only significantly enriched in the genes down-regulated in *rx3*^*-/-*^ mutants (q < 0.05). More detailed information about the deregulated genes was obtained by using more refined GO terms (Figure 3E, F). For genes down-regulated in *rx3*^*-/-*^ mutants, the most significantly enriched terms include “*regulation of transcription”* (*e.g. rx2*, *rx3*, *six7*, *rorab*, *vsx2*, *foxd1*, *six6b*, *rorb*, *hmx1*), “*eye development and morphogenesis”* (*e.g. rx3*, *mab21l2* and *six7*) and “*neural crest cell migration”* (*e.g. rx3*, *foxd1*). For genes up-regulated in *rx3*^*-/-*^ mutants, terms associated with brain development are significantly enriched, including “*telencephalon development”* (*e.g. dmrta2*, *emx3*) and “*forebrain development”* (*e.g. fezf2*, *lhx5*). These gene ontologies are consistent with the phenotype of *rx3*^*-/-*^ mutants which fail to complete optic vesicle morphogenesis and exhibit expanded forebrains.

In parallel, KEGG pathway annotation was used to profile signaling pathways associated with the differentially expressed genes [29]. Genes down-regulated in *rx3*^*-/-*^ mutants are associated with circadian rhythms (*e.g. rorb*, *rorab*), and metabolism (*e.*g. *adh8b*, *aldh1a3*) (Figure 3G, H). Genes up-regulated in *rx3*^*-/-*^ mutants are associated with p53 signaling (*e.g. igfbp3*, *bai1*), axon guidance (*e.g. ntn1b*, *ppp3ca* and *dpysl2*), and Wnt and Hedgehog signaling (*e.g. wnt7ba*, *wnt7bb* and *ppp3ca*). Wnt, Hedgehog and p53 signaling pathways are crucial for brain development [30, 31]. Previous reports hypothesize that Rx3 prevents retinal progenitor cells developing into forebrain cells by inhibiting Wnt signaling [11, 32, 33]. Here, demonstration that elevated expression of Wnt signaling genes occurs in *rx3*^*-/-*^ mutants, during optic vesicle morphogenesis, supports this hypothesis.

### Co-expression network of Rx3-associated genes

**Figure 4:**
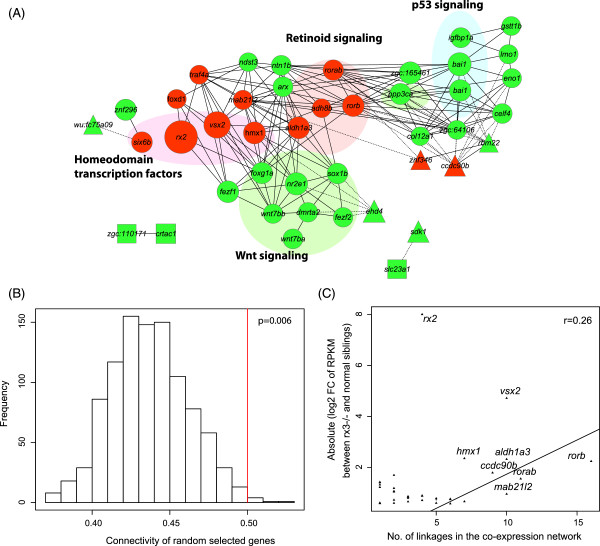
**A gene co-expression network of** ***rx3*** ^***-/-***^ **regulated genes. (A)** In red and green, are the gene nodes down-regulated or up-regulated, respectively, between *rx3* ^*-/-*^ mutants and siblings. Group 1, 2 and 3 genes from the hierarchical clustering analysis in Figure 5 are represented by colored circles, rectangles or triangles, respectively. Edges connecting different nodes indicate co-expression (correlation coefficient r ≥ 0.95 or ≤ -0.95) between genes, with solid lines for positive correlations and dashed lines for negative correlations. **(B)** Distribution of the connectivity scores from the permutation depicted as a bar plot. The connectivity score was calculated as the average absolute correlation coefficient of any gene pairs in the selected genes. The permutation test was performed 1000 times on randomly selected genes. The red line is the connectivity score of *rx3* ^*-/-*^regulated genes. **(C)** A dot plot showing that the fold change in expression of deregulated genes between *rx3* ^*-/-*^ mutants and siblings positively correlates with the number of gene linkages on the co-expression network. A fitted linear model of fold changes to the number of linkages was plotted. Genes with a higher number of connections in the co-expression network have higher fold changes in *rx3* ^*-/-*^ mutants.

**Figure 5:**
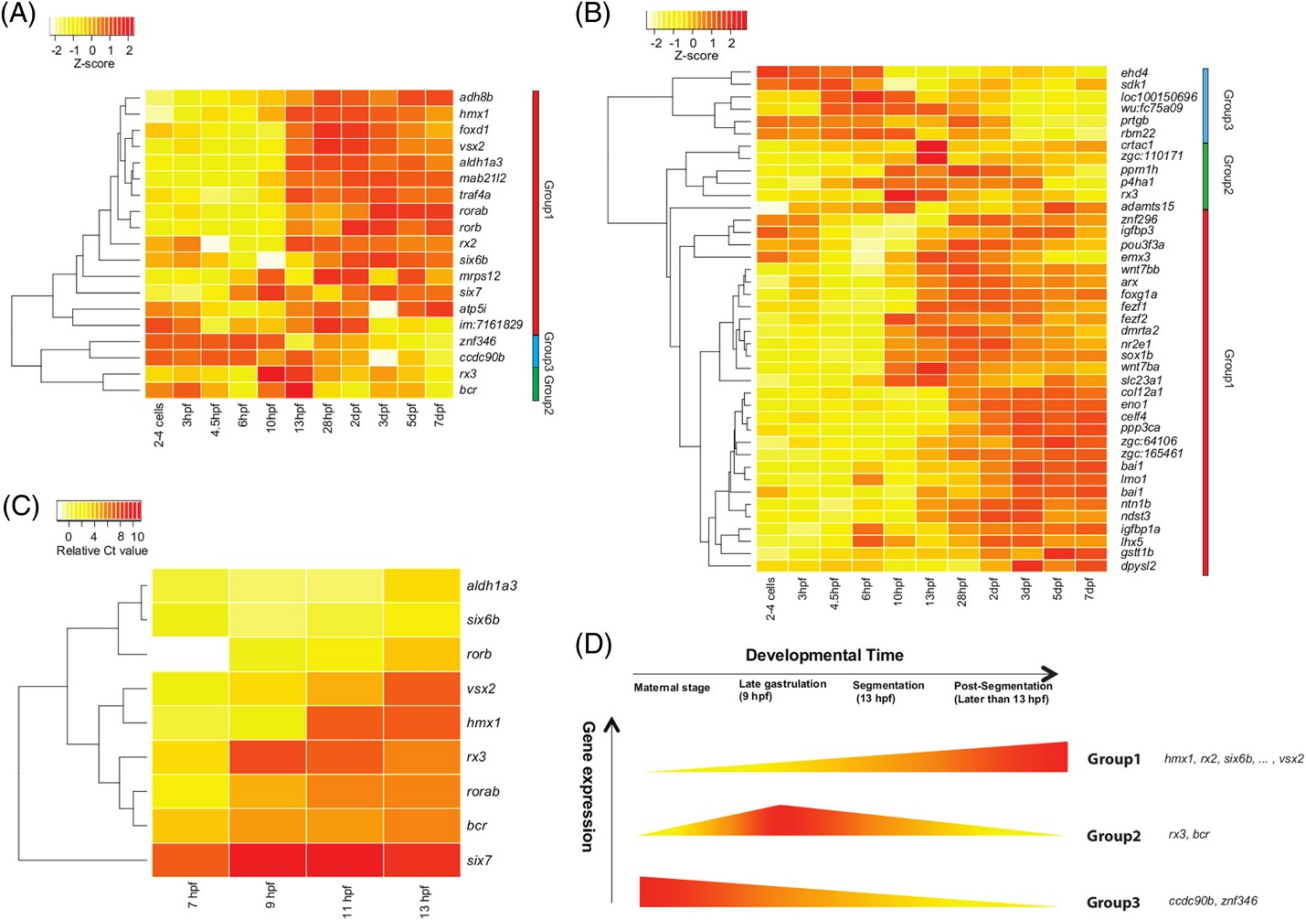
**Synchronous temporal expression profiles of Rx3-regulated genes. (A-C)** Heatmaps depicting the temporal expression profile of Rx3-regulated genes clustered hierarchically. Hierarchical clustering based on the z-scores of relative RPKM values for *rx3* ^-/-^ down-regulated **(A)** and up-regulated **(B)** genes. Genes were classified into 3 groups showing different synchronous temporal expression profiles. **(C)** Hierarchical clustering based on real-time PCR data. **(D)** Schematic view of progressive expressions of Rx3-regulated genes during early eye development. *rx3* has a peak of expression at 9 hpf, and most of the Rx3-regulated genes show a transition of expression after that. Most of the genes exhibit increased expression after the onset of *rx3* (Group 1). A few genes exhibit decreased expression after the onset of *rx3* (Group 3).

Pathway annotations provided by public databases are incomplete and not suitable to build networks regulated by a single transcription factor. This greatly limits our understanding of the signaling network regulated by Rx3. However, gene co-expression network is a powerful tool to profile functional relevance between genes, which can provide linkage of genes based on temporal or spatial gene expression patterns. We constructed a gene co-expression network using the developmental expression profile of Rx3 regulated genes during zebrafish early development. The developmental stages span from maternal stages to larval stages with matured visual function, including wildtype larva at 13 hpf from our RNA-seq data and publicly available RNA-seq data sets from zebrafish developmental stages of 2–4 cells up to 7 dpf [34, 35] (Figures 4 and 5).

Linkage of genes was decided using correlation scores of gene expression during early development. Genes with correlation coefficients of ≥0.95 or ≤ -0.95 were connected in the network to indicate significant gene co-expression (Figure 4A). Genes within the same signaling pathway tend to connect with each other or through a common mediator. These connected genes form distinct groups in the co-expression network. Homeodomain transcription factors and genes related with retinoid-signaling emerged as the two major hubs for genes down-regulated in *rx3*^-/-^ mutants. The expression of other down-regulated genes, including *mab21l2*, *foxd1* and *tra4a* highly correlate with the homedomain transcription factors and expand from this hub. The Wnt signaling pathways emerged as the major hub for genes up-regulated in *rx3*^-/-^ mutants. The KEGG pathway annotation only associated *wnt7ba*, *wnt7bb* and *ppp3ca* with the Wnt signaling pathway. By interrogating the co-expression network and the literature, the Wnt signaling pathway modulated in *rx3*^-/-^ mutants was extended to include *dmrta2* [36], *fezf2* [37], *nr2e1* [38], *sox1b* [32], *foxg1a* [39] and *emx3* [11, 40].

To assess the connectivity of the genes deregulated in *rx3*^-/-^ mutants, we performed a permutation test against randomly selected genes. The connectivity score is calculated as the average absolute value of correlation coefficient of any gene pairs in the selected gene set. Genes deregulated in *rx3*^-/-^ mutants showed significantly higher connectivity (connectivity score 0.50) over randomly selected genes (Permutation p = 0.006) (Figure 4B).

The co-expression network indicates that Rx3 regulates optic vesicle morphogenesis through a complicated and highly modulated gene network. Genes in the center of such networks are usually coupled with more linkages to other genes in the network. Interestingly, the number of linkages shows a positive correlation with the fold change of gene expression between *rx3*^-/-^ mutants and wild-type phenotype siblings (correlation coefficient r = 0.26) (Figure 4C). A plausible explanation is that Rx3 has a higher impact on genes in the center of the network (*e.g. aldh1a3*, *mab21l2*, *rorb*, *rorab* and *vsx2*), and then extends its regulation though these genes to the periphery of the network.

### Temporal expression of Rx3-associated genes

The high connectivity of Rx3 regulated genes in the co-expression network indicates highly correlated gene expression during early eye development. However, little is known about the developmental expression of *rx3* or temporal associations with its gene targets during optic vesicle morphogenesis. Thus we investigated the temporal expression profiles of the Rx3 associated genes using the aforementioned developmental data.

The timely onset of Rx3 is required to ensure optic vesicle separation from the telencephalon at the late gastrulation stage [11]. As expected, the expression of *rx3* peaks at late gastrulation and early-segmentation stages (~9 to 13 hpf) when optic vesicles start to enlarge (Figure 5A). Most of the genes deregulated in *rx3*^*-/-*^ mutants exhibit a modulation of gene expression levels after the onset of *rx3*. Hierarchical clustering analyses were performed to group the genes by temporal expression patterns. Genes were classified into three groups based on temporal expression correlations with *rx3* (Figure 5A-D). *Group 1* genes exhibit increasing expression after the onset of *rx3* expression at 10 hpf. *Group 1* includes the highest ranking down-regulated genes, such as the homeodomain transcription factors genes *rx2, six6b*, *six7, hmx1* and *vsx2,* the retinoic acid-related receptor genes *rorb* and *rorab*. Up-regulated genes such as the Wnt signaling genes; *wnt7ba, wnt7bb* and *ppp3ca,* and p53 signaling pathway genes; *igfbp3* and *bai1,* also belong to *Group 1. Group 2* genes show additional temporal correlations with *rx3* expression, having a waveform pattern peaking at 10–13 hpf. *Group 2* includes the GTPase-activating protein, *bcr* (correlation coefficient r = 0.64). This synchronous pattern of gene expression indicates gene interaction, but to date no direct regulation of the *Group 2* genes by Rx3 has been reported. *Group 3* genes including *ccdc90b*, *znf346, sdk1 and ehd4* show decreasing expression after 10 or 13 hpf *i.e*. after the onset of *rx3*.

To apply an independent temporal analysis of *rx3*-regulated genes, real-time PCR was performed on selected genes from 7 to 13 hpf (Figure 5C). The expression of *rx3* peaked at 9 hpf when the neural plate forms [41] and is slightly reduced at 11 and 13 hpf, consistent with the RNA-seq observations (Figure 5A). Most genes (*e.g. bcr*, *hmx1*, *rorb*, *rorab*, *six7* and *vsx2*) show gradual increases and higher expression after the onset of *rx3* at 9 hpf, consistent again with the RNA-seq results (Figure 5A, C).

In summary, a majority of the analysed genes showed altered temporal gene expression around 9–13 hpf after the onset of *rx3* at ~9 hpf. This indicates that the onset of *rx3* is critical to ensure accurate expression at the stage when optic vesicles form. The analyses revealed cohorts of genes that i) are reduced in 13 hpf *rx3*^*-/-*^ mutants and whose expression is normally switched on after the onset of *rx3* expression (*e.g. rx2, six6b*, *six7*); or ii) are increased in 13 hpf *rx3*^*-/-*^ mutants and whose expression normally is switched off during the onset of *rx3* expression (e.g. *wnt7bb*, *foxg1a*, *igfbp3*). These cohorts represent genes putatively activated or repressed in a progressive specification manner by Rx3 (Figure 5D).

### Identification of consensus Rx3-binding sites in differentially expressed genes

**Figure 6:**
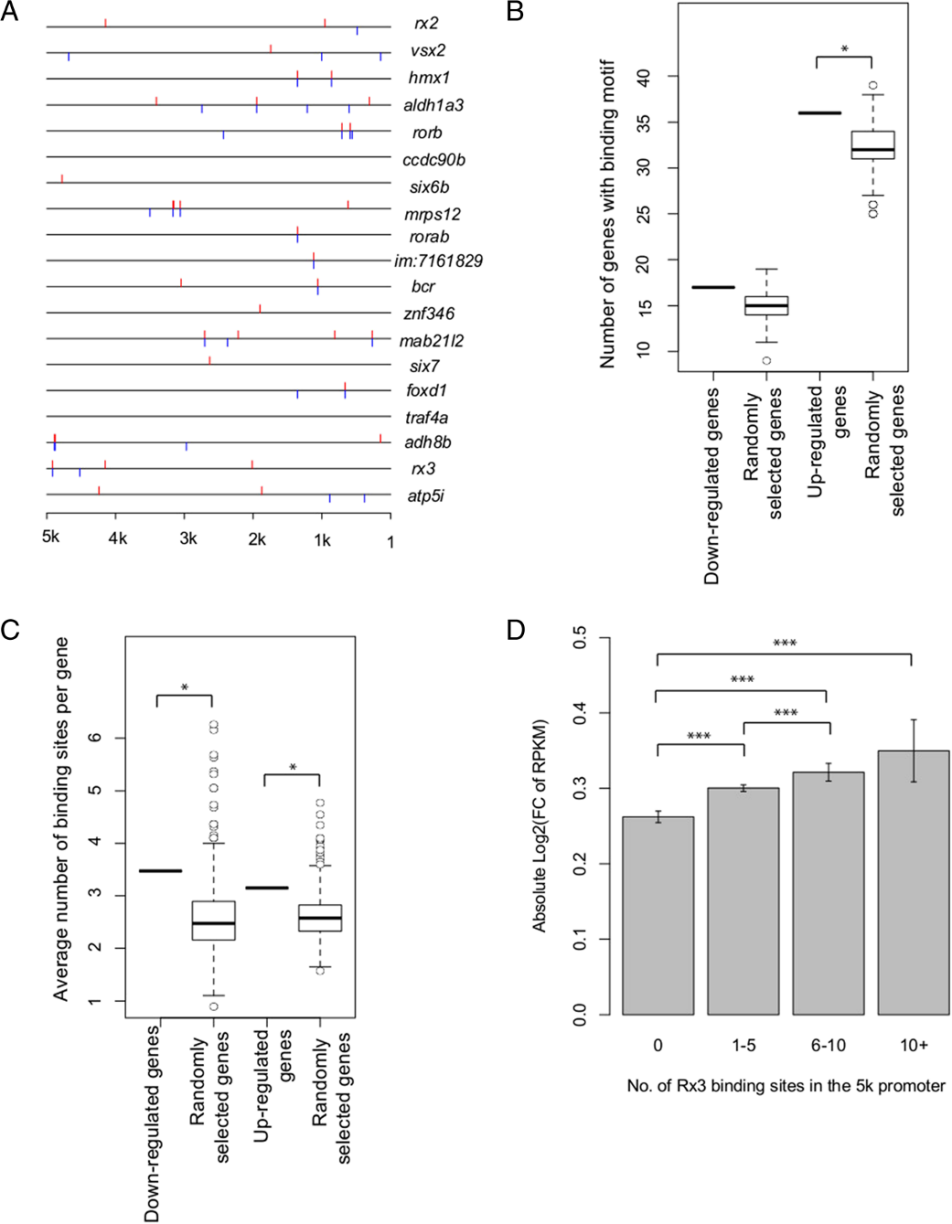
**Presence of RAX-binding sites in** ***rx3*** ^***-/-***^ **deregulated genes and effect on gene expression. (A)** RAX-binding sites in the 5 kb upstream promoter regions of the *rx3* ^*-/-*^ down-regulated genes. Red bars are consensus binding sites on the positive strand, while blue bars are consensus sites on the negative strand. The enrichment of RAX-binding sites in the promoter regions of deregulated genes was evaluated by 1000 times permutation. The number of random shuffled genes with RAX binding sites **(B)** and the frequency of RAX-binding motifs in the promoter regions **(C)** are plotted. *, p < 0.1. **(D)** Absolute log2 transformed gene expression fold changes between *rx3* ^*-/-*^ mutants and wild-type phenotype siblings were calculated for genes with diferent number of RAX-binding sites on 5 kb promoter. Significance between different groups of genes was evaluated using one-tail Wilcoxon rank sum test. ***, p < 0.001.

To support the contention that the deregulated genes in *rx3*^*-/-*^ mutants are potential Rx3 targets, we evaluated the presence of Rx3-binding motifs in promoter regions of the deregulated genes [8]. The presence of consensus binding motifs for RAX, an *rx3* ortholog in humans, was sought in 5 kb promoter regions of selected zebrafish genes. The RAX-binding motif has a conserved 5′-TAATTA-3′ sequence in the center. Importantly, RAX-binding motifs were identified in the promoter regions of 17 of the 19 genes significantly down-regulated in 13 hpf *rx3*^*-/-*^ mutants (Figure 6A). RAX binding sites were also identified in 36 out of the 40 genes up-regulated in *rx3*^*-/-*^ mutants.

To estimate the probability of identifying RAX-binding sites randomly, we compared the enrichment of RAX-binding sites against 1000 times randomly selected genes. Both the proportion of genes with RAX-binding motifs and the number of RAX-binding motifs in the 5 kb promoter regions were determined in the deregulated genes and randomly selected genes (Figure 6B-C). A significantly higher proportion of genes with RAX-binding motifs were identified in the *rx3*^*-/-*^ up-regulated genes compared to the randomly selected genes (Permutation p < 0.1) (Figure 6B). Furthermore, significantly higher numbers of RAX-binding motifs were identified in the 5 kb promoter regions of both down- and up-regulated genes (Permutation p < 0.1) (Figure 6C). The presence of multiple binding sites for the same transcription factor can facilitate regulation of gene expression by increasing transcription factor binding affinity and providing functional redundancy [42]. Thus, we further investigated the effect of number of multiple RAX-binding sites on the gene expression changes in the *rx3*^*-/-*^ mutants. Genes with higher number of RAX-binding motifs exhibited larger gene expression changes in the *rx3*^*-/-*^ mutants (one-tailed Wilcoxon rank sum p < 0.001) (Figure 6D). In summary, this analysis suggests genes significantly deregulated in *rx3*^*-/-*^ mutants are mostly enriched with multiple consensus Rx3-binding sites and potentially actively regulated by Rx3 at 13 hpf.

### Model of Rx3 regulation during early development

**Figure 7:**
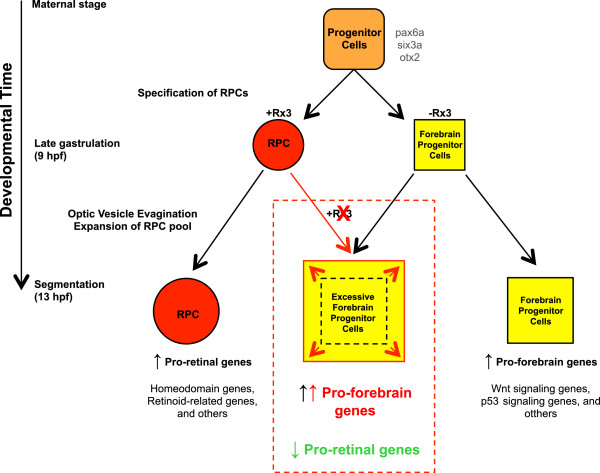
**A proposed model of the role of Rx3 in early eye development.** Rx3 functions to up-regulate pro-retinal genes and down-regulate pro-forebrain genes in the wild-types. Without the function of Rx3 (as indicated in the red dotted box), pro-retinal genes are not up-regulated and pro-forebrain genes are not repressed leading to a failure to properly form eyes and an expanded forebrain.

Based on the above analyses, we assembled a model of zebrafish Rx3 regulation during early eye development (Figure 7). Neural tissue which expresses *pax6a*, *six3a* and *otx2* progresses to form retinal progenitor cells (RPCs) or forebrain progenitor cells [13]. These genes are not significantly deregulated in *rx3*^*-/-*^ mutants. In the absence of Rx3, the optic vesicles do not evaginate, the RPC pool does not expand and consequently RPCs become forebrain cells [6, 11–13]. Our evidence supports the view that this default outcome results from enhanced expression of pro-forebrain genes that are normally repressed by Rx3. In addition, there is reduced expression of pro-retinal genes normally activated by functional Rx3. Our results support a model of progressive specification following Rx3 mediated up-regulation of homeodomain transcription factors and retinoid-signaling pathways for eye development, and down-regulation of pro-forebrain Wnt signaling pathways.

## Discussion

Here we report, whole transcriptome sequencing of zebrafish *rx3*^*-/-*^ eyeless mutants, a model of human anophthalmia [43]. Rx3 is a conserved transcription factor, whose orthologues are required for eye development in all vertebrates examined, including fish, frogs, mice and humans [2, 3, 9, 23, 44–46]. Here, we show that genes previously linked with microphthalmia, including *aldh1a3*, *rx2*, *six6b*, *vsx2*, and recently *mab21l2* with anophthalmia [47], are constitutive elements of an Rx3-regulated gene network (Table 2, Figure 4). This *proof-of-principle* supports the contention that additional members of this network are candidate genes for congenital defects in human and animal eye development. In addition, these genes represent therapeutic targets as Rx genes can also promote retinal regeneration [48]. Indeed, Rx-regulated genes can be applied to cell-based therapies, directing stem cells to retinal progenitor fates prior to transplantation [49].

### Identification and validation of novel Rx3-regulated genes

Here, whole transcriptome mRNA sequencing (RNA-seq) was used to profile global gene expression changes in 13 hpf *rx3*^*-/-*^ mutants. This stage of eye development was selected as the earliest time point that optic vesicle evagination phenotypes could be detected reliably, and because it is only several hours after the onset of *rx3* expression in the eye field [19]. This experimental design was selected to concentrate analyses on the network of genes that are de-regulated shortly after the absence of functional Rx3 and not genes absent due to the later eyeless phenotype. For example, *nr2e1* regulates later stages of eye development, consistent with its expression in brain regions at the 13 hpf optic vesicle stage and subsequent expression in the eye field from 19 hpf [50, 51]. We compared gene expression profiles of *rx3*^-/-^ mutants versus wild-type phenotype siblings to identify potential Rx3 regulated genes. We considered wild-type phenotype siblings as a better control than wild-type AB strains, because both *rx3*^-/-^ mutants and siblings originate from the AB zebrafish strain (*chk*^*w29*^ allele) by a mutagenesis screening. Our preliminary analyses suggest that normal phenotype siblings and *rx3*^-/-^ mutants share >27,000 single nucleotide variations which are not found in wildtype AB fish (data not shown). This supports the appropriateness of comparing siblings to mutants.

Using whole embryos in the gene expression comparison offers a significant advantage in terms of consistency of tissue sampling to produce less varied data. However, if genes are expressed extensively in fields other than the morphologically affected tissues in *rx3*^*-/-*^ mutants (i.e. outside of eye or forebrain fields), differential changes in expression may be not be sufficiently detectable by RNA-seq. Extensive expression domains may explain why some known differentially expressed genes in *rx3*^*-/-*^ mutants identified by *in situ* hybridization were not confirmed by our whole transcriptome approach (such as *epha4a* or *ephb4a*) [18, 52, 53]. However, we did observe deregulation of some genes with known extensive expression domains (e.g. Table 1, netrin 1 [54, 55]).

Following bioinformatic analysis of the RNA-seq data, sixty-nine genes, or ~0.2% of known zebrafish genes, demonstrate a 1.5-fold or higher change in gene expression between 13 hpf wild-type phenotype siblings and *rx3*^*-/-*^ mutants (Figure 1). These differentially expressed genes represent both direct and indirect targets of Rx3. The presence of RAX-binding motifs as defined by a position weight matrix in the de-regulated genes was shown to further support the contention that some of the genetic network is directly regulated by Rx3 [8, 56]. The core of the RAX binding motif is a short sequence “TAATTA”, thus it is relatively common to find this sequence in the zebrafish genome (about 1 in every 4 kb). In order to estimate the possibility of finding RAX motifs by chance in the Rx3 regulated genes, a permutation test was performed. Indeed, an enrichment of putative RAX-binding sites was demonstrated in genes significantly deregulated in 13 hpf *rx3*^*-/-*^ mutants (Figure 6B). Genes with higher number of RAX-binding sites on promoter are more likely to be actively regulated by Rx3, and exhibit larger change of gene expression in the *rx3*^*-/-*^ mutants (Figure 6C, D). Rx binds to what is referred to as the PCE-1 element (photoreceptor conserved element-1), which is based on the TAAT homeodomain binding core sequence, in photoreceptor promoters to enhance gene expression [57–61]. The lack of a zebrafish Rx3-specific antibody hinders direct validation of these binding sites in the context of optic vesicle morphogenesis *in vivo*.

Quantitative real-time PCR (qRT-PCR) and/or *in situ* hybridisation validated the differential expression of *mab21l2, rx2, hmx1, six6b, vsx2, aldh1a3* and *rorb* (Figure 2). These results highlight the accuracy and sensitivity of the RNA-seq dataset, which is comparable to qRT-PCR, the current standard quantitative method for profiling gene expression. Most of the Rx3-regulated eye field genes were only partially down-regulated, their residual expression being likely due to expression in domains outside of the eye field or partially reduced expression in the eye field due to redundant factors [12]. For example, *mab21l2* is not expressed in *rx3*^*-/-*^ mutant optic vesicles, but its expression in non-eye tissues (e.g. tectum) is unaffected, *i.e.* Rx3-independent [13]. Similarly, *rorab* has a partial down-regulation in *rx3*^-/-^ mutants but is also expressed in tissues beyond the eye [50]. Interestingly *rx1,* which is expressed solely in the eye, is only slightly down-regulated in *rx3*^*-/-*^ mutants. This indicates that alternative regulators can remain in the eye field to specify retinal cells, but that *rx3* is required for proper optic vesicle morphogenesis. Indeed, we confirmed that several other genes enriched for optic primordium expression, were not significantly deregulated in *rx3*^*-/-*^ mutants (*e.g. arhgap32*, *exoc6, lrrtm1* and *swap70*) (Figure 2A, B) [27, 28]. In summary, these results support our conclusion that the 69 genes differentially expression in 13 hpf *rx3*^*-/-*^ mutants are indeed constituents of a genetic network deregulated shortly after the absence of Rx3 and not merely due to the morphological absence of the eye.

### Rx3-regulated genetic network during early development

The recessive *rx3* mutation causes an eyeless phenotype along with an expanded telencephalon [11, 13]. These morphological phenotypes are consistent with the molecular profile of gene set enrichment using GO and KEGG pathway databases. Genes down-regulated in *rx3*^*-/-*^ mutants are mainly involved in eye development, and the up-regulated genes are mainly involved in brain development (Figure 3). Specification of the eye field is controlled by a network of eye field transcription factors. The co-expression network built in this study provides insights to Rx3 directed gene interaction during early eye development. Genes deregulated in the *rx3*^*-/-*^ mutants show high levels of connectivity to each other (Figure 4B). Several homeodomain transcription factors (e.g. *hmx1*, *rx2*, *vsx2*) and retinoid-signaling genes (e.g. *aldh1a3*, *rorab*, *rorb*) are at the center of the network. These genes tend to change more dramatically in the absence of Rx3, thus are more highly regulated by Rx3 (Figure 4C). Through these hub genes, Rx3 may act indirectly to regulate more peripheral genes in the network [62]. Homeodomain transcription factors have been shown to be expressed in the anterior region of the neural plate in vertebrates and have a dynamic, overlapping pattern of expression in the presumptive eye field in *Xenopus laevis* [12] *.* They form a self-regulating feedback network that specifies the vertebrate eye field and include *rx1, pax6, six3, lhx2, nr2e1 (tll)* and *six6 (optx2)*. Our analyses indicate that at least one paralog of all of these genes are deregulated in 13 hpf *rx3*^*-/-*^ mutants, e.g. *rx2*, *pax6b, six3b, lhx2, nr2e1* and *six6b* are deregulated, whereas their paralogs do not show differential expression. Similarly, at this timepoint, the *rorα paralog b*, but not *paralog a*, is deregulated in *rx3*^*-/-*^ mutants (Additional file 2: Table S1). Thus, Rx3 differently regulates the expression of transcription factor paralogs indicating a sub-functionalization of the regulation and expression of these gene paralogs [63].

Structural eye malformations can be due to either an excess or deficiency of retinoids. Here, we show that Rx3 is required for normal expression levels of retinoic acid receptor related (RAR)-orphan receptors. Although referred to as orphan receptors, evidence supports *rorb* binding retinoic acid [64]. Retinoic acid is derived from vitamin A by sequential oxidation steps, the generation of retinaldehyde by retinol dehydrogenases (RDH) and the synthesis of retinoic acid by retinaldehyde dehydrogenases (RALDH) [65]. One of the two zebrafish *raldh* genes, *aldh1a3*, also has reduced expression in *rx3*^*-/-*^ mutants. This is consistent with the known role of retinoic acid in eye development including ano-/microphthalmia in humans and zebrafish due to defects in retinoic acid supply [66]. For example, drug inhibition of the *aldh1a3* enzyme produces zebrafish with a small eye phenotype [66, 67]. We propose that Rx3 is priming retinal progenitor cells to be responsive to retinoids including retinoic acid or other unidentified ligands for the subsequent optic cup stage of eye development. Loss of forebrain and eyes is most frequently observed upon overactivation of the Wnt/beta-catenin pathway [68–72]. Wnt-signaling deregulation has been described previously in *rx3*^*-/-*^ mutants, including up-regulation of both known positive and negative factors that affect Wnt signaling (*emx3* and *foxg1* respectively) [11, 15, 40, 73]. Our data expands on this to a larger cohort of upregulated genes that may lead to augmented Wnt signaling in parts of the forebrain, including the expanded telencephalon or diencephalon regions [11]. As an example from our extended cohort, Wnt7ba/bb are reported to be expressed in diencephalon-telencephalon border at the 10-somite stage [40, 74].

Temporal expression profiling of genes significantly deregulated in *rx3*^*-/-*^ mutants revealed clusters of genes with synchronised temporal expression patterns (Figure 5). The majority of genes show altered gene expression profiles around the peak of *rx3* expression, confirming its critical role in ensuring accurate expression at this stage of eye development. By examining public *in situ* hybridisation images, the earliest time points of robust detectable expression of *rx2* [19], *hmx1* [75], *rorab* [76] and *rorb* [53] are 10, 14, 12 and 16 hpf respectively, a few hours after the onset of *rx3* [19, 23, 77]. *six7* begins its expression as early as 6 hpf [78]. These observations are very consistent with the data from real-time PCR and RNA-seq in this study (Figure 5A, C). These genes gradually increase after the peak of *rx3* expression, indicative of more downstream roles of Rx3 regulation.

## Conclusion

In summary, Rx3 functions during the optic vesicle stage of development to enhance expression of transcription factors, in particular homeodomain genes, and retinoid-signaling genes, while also inhibiting brain specification pathways. Genes deregulated in the *rx3*^-/-^ mutants showed enrichment of multiple RAX-binding motifs and high gene co-expression connectivity. We propose a model of eye development based on a complex gene network which allows for progressive steps of specification. This Rx3-regulated gene network supports a hierarchical expression of eye field transcription factors specifying early eye development.

## Methods

### Zebrafish husbandry and sample collection

Zebrafish were handled according to standard protocols and animals studies were approved by the University College Dublin Animal Research Ethics Committee (AREC-P-08-54). Heterozygous carriers of a recessive *rx3* mutation (*chk*^*w29*^ allele) were mated. These fish originate from a mutagenesis screen performed in AB zebrafish as described in [13], and have an equivalent mutation as to *chk*^*s399*^ mutants [9] which were further described in [11]. This mutation results in a premature stop codon at amino acid Y133 (Y133X) in the DNA-binding homeodomain of the *rx3* gene. Pairwise matings were used to generate embryos which were pooled prior to sample collection. Offspring were grown to the 8-somite stage in embryo medium containing methylene blue at 28.5°C in 10 hour dark: 14 hour light cycle conditions, where the 8-somite stage is equivalent to 13 hpf under these conditions [79]. The eyeless mutant (*rx3*^*-/-*^) and eyed sibling (morphologically wild type siblings consisting of an unknown ratio of wild-type *rx3*^*+/+*^ and heterozygous *rx3*^*+/-*^ carriers, herein referred to as ‘normal or wild-type phenotype siblings’) samples were distinguished based on their morphology using a dissecting light microscope at the 8-somite stage. Three biological replicates were collected for each sample and one replicate of wild-type (AB strain) embryos at the same developmental stage was collected. Each replicate contained pools of 10 whole embryos. Samples were collected in RNA*later* (Qiagen, Hilden, Germany) and stored at 4°C until processing. The RNeasy mini RNA extraction kit was used to isolate RNA, on-column DNaseI digestion was performed and RNA was collected in RNase-free water, as per manufacturer’s instructions (Qiagen, Hilden, Germany). A Nanodrop spectrophotometer (Beckman, USA) was used to determine the concentration of RNA and a Bioanalyser (Agilent, Santa Clara, USA) was used to confirm the RNA Integrity Number (RIN) of the samples as being between 8 and 8.8.

### cDNA library generation and sequencing

Using 1 μg of total RNA, cDNA libraries were prepared using the mRNA-seq 8-Sample Prep Kit as per manufacturer’s instructions (Illumina, RS-100-0801). Briefly, poly-A containing mRNA molecules were purified using poly-T oligo-attached magnetic beads. Following purification, mRNA was fragmented to ~200 base pair fragments and cDNA generated with ligated adaptors. These products were purified and PCR enriched to create the final cDNA library. Single-Read Cluster Generation Kit v4 was used for cluster generation as per manufacturer’s instructions (Illumina, GD-103-4001). Briefly, cDNA library samples were bound to complementary adapter oligonucleotides grafted on the surface of the Illumina Genome Analyzer flow cell. The templates were copied from the hybridized primer by 3′ extension using a high fidelity DNA polymerase. The 36 Cycle Sequencing Kit v4 on the Genome Analyzer II X platform was used for sequencing (FC-104-4002). The phi X 174 (PhiX) bacteriophage genome DNA was used as a control lane to validate sequencing quality. Samples were sequenced to 42 bp. After 40 bp the sequencing quality at 3′ ends significantly decreased under the acceptable threshold of 20 and sequencing reads were therefore trimmed to 40 bp to ensure acceptable quality. RNA-seq data sets were uploaded to GEO with a series accession number GSE52652.

### Reads alignment and genome annotation

RNA-seq reads were mapped to the zebrafish genome version 9 using TopHat [80] allowing 2 mismatches. TopHat can detect reads across exon splice junctions. Novel transcripts were assembled by Cufflinks using mapped reads [81]. Novel transcripts less than 300 bp were discarded. A customized transcriptome was built by integrating known transcripts from RefSeq, Ensembl and GenBank (downloaded from the UCSC genome browser, February, 2011) [82] and novel transcripts assembled by Cufflinks. These transcripts were clustered into genes based on coding sequence (CDS) evidence using a revised protocol which we previously proposed [21]. Firstly, transcripts with known CDS were clustered into genes by overlapping CDS. Then, transcripts without CDS definitions were clustered into these defined genes if they overlapped with these genes in transcribed regions. Transcripts without overlap to known genes were classified as novel genes. A total of 31,731 genes were defined using the combined set of transcripts. In order to predict function for novel genes, nucleotide sequences of the novel genes were searched against the NCBI nr database using BLASTX [83].

### Counting reads and statistical analysis of RNA-seq data

RNA-seq reads were counted using HTSeq [84]. Only genes having greater than 10 reads from replicate samples progressed to statistical analysis, leaving 21,267 genes. Gene expression level was calculated using the normalized value: reads per kilobase per million reads (RPKM) [85]. Differentially expressed genes were selected using the Bioconductor package DEGSeq based on read counts following a Poisson distribution model [86]. P-values from DEGSeq were corrected using the Bonferroni method. Differentially expressed genes were selected as fold change ≥ 1.5 or ≤ 2/3 with corrected p-value <0.001. Gene set enrichment analysis was performed for differentially expressed genes based on Fisher’s Exact Test. Gene Ontology (GO) [87] and KEGG pathway annotations [29] were downloaded for gene set annotation. In order to improve the zebrafish pathway annotation, KEGG pathway annotations of human homologs were combined with zebrafish annotations for pathway analysis. To study the association of zebrafish genes with human inherited diseases, NCBI OMIM databases were downloaded and human diseases with known causative genes were associated with their zebrafish homologs.

### Identification of RX transcription factor binding motifs

A human RAX position weight matrix was downloaded from MatInspector (Genomatix, Germany). The core of the RAX binding motif is a short sequence “TAATTA”. 5 kb promoter sequences were downloaded for zebrafish genes using the UCSC genome browser [82]. Consensus RAX binding sites were searched for on both strands of promoter sequences using Possumsearch with a threshold of 80% matrix similarity [88]. In order to estimate the possibility of identifying the binding motifs randomly, permutations were performed by randomly selecting the same number of genes as the differentially expressed genes 1000 times. RAX binding sites were searched on the 5 kb promoter sequences of these randomly selected genes. P-values were obtained by counting the chance of obtaining equal or higher number of genes with RAX motifs on the promoter, or equal or higher number of motifs per gene promoter out of 1000 permutations.

### Quantitative RT-PCR

cDNA was synthesised from independent biological replicates of 8-somite stage mutants and siblings by reverse transcription after priming with random hexamers using the Invitrogen Superscript III system. 200 – 500 ng RNA was used per sample. Real-time PCR was performed using Taqman probes as the reporter in the 18S rRNA control samples and SYBR Green as the reporter in all other reactions. Primer sequences for *aldh1a3*: forward GCCCATCGGTGTGTGCGGAG; reverse GGGTCTGCTCCGCCGGTTTG, for *bcr*: forward GCGTCCGGAGCGTGCAGAGTG; reverse GAGGCAACTGATGGACCGTCTGGAG, for *exoc6*: forward TGAAAACCATGGAGCAGCTA; reverse CTGTGCTTAAGCCGTTGTGA, for *hmx1*: forward AGCGGTTGTGCGGTTGACGA; reverse GGCTGAGCCGGGTTCGGAAG, for *im:7150060/arhgap32*: forward CCAGTTGCTTGTGATTAAAACCT; reverse AAGCCCAGTCAGTGCAACTT, for *lrrtm1*: forward AACACCCTTGAGTGGACCTG; reverse GTGTGGTTGCTTCCACATTG, for *mab21l2*: forward TCGGGCTGTAGGAAGAAATG; reverse TCTCTTGCCAGTCTCCAGGT, for *rorab*: forward TTAGCAGTGGGCATGTCAAG; reverse GAGGGCTGAATGTCCAGGTA, for *rorb*: forward AAGCAGAAGCCCTCGCTCGGG; reverse CACCGTTCGCCTGGCCCTTG, for *rx2*: forward CGATGCAGATTTGGGAGAC; reverse AGGCAGGTTGACTTTCATGG, for *rx3*: forward GTGGCCTGCCGTTAGAGCCC; reverse CGGCCTGCTGAGGGGTGATG, for *six6b*: forward TCGAACGGCTCGGTTCGGTC; reverse GCGGCTTTGCTGGACAGGGCT, for *six7*: forward GGCGGGAATTTCGAGGCGCT; reverse GCTCCGCCTCCCGGTAATGC, for *swap70*: forward CTGCTGGAGGAGGAAGTGTC; reverse CTGAATACTGAGAAAAGATCATCACC, for *vsx2*: forward AACGGGGGAAATAACAATCC; reverse CTGAGCTGGCAGACTGGTTA. A eukaryotic 18S rRNA probe (Applied Biosystems, 4310893E) served as an internal reference for normalisation. The initial cycle was 2 minutes at 50°C and 10 minutes at 95°C. Then the samples were cycled at 95°C for 15 seconds and 60°C for 1 minute. Ct values were normalized according to the lowest abundant sample. Student’s t-test was used to determine significance with a p-value <0.05.

### Wholemount *in situ*hybridisation

Expression of zebrafish genes (*hmx1* and *six7*) was analyzed in *rx3*^-/-^ mutants (*chk*^*w29*^ larvae) by wholemount *in situ* hybridization. *six7* cDNA-containing plasmid was a gift from Professor Hee-Chan Seo’s lab [78]. *hmx1* cDNA-containing plasmid was a gift from Professor Andrew J. Waskiewicz’s lab [75]. These plasmids were used to generate DIG-labelled probes. Embryos were fixed overnight at 4°C in 4% paraformaldehyde (PFA). *In situ* hybridization was performed on whole wild-type and mutant embryos as previously described [89].

### Public zebrafish development RNA-seq data sets

Public zebrafish RNA-seq data sets from early developmental stages were downloaded from GEO with accession number GSE22830 [34] and Sequence Read Archive ERP000400 [35]. RNA-seq reads were aligned using Tophat [80] using the customized transcriptome built in this study as described above. RNA-seq reads were counted using HT-seq. RNA-seq data for wild type larva at 13 hpf from this study was incorporated with the public data sets. Read counts were transformed into RPKM values and quantile normalized. Biological replicates from the same developmental stages were averaged. Hierarchical clustering was performed using Euclidean distance with complete linkage. Gene co-expression groups were defined by cutting the hierarchical clustering trees.

### Constructing a gene co-expression network

Gene correlation scores were calculated using normalized log2 transformed RPKM values from the public RNA-seq data sets and 13 hpf wild type larvae produced in this study. Correlation coefficients ≥0.95 or ≤ -0.95 were selected as showing high positive and negative co-expression. Genes showing high co-expression with at least one other gene were included in the co-expression network. The co-expression network was plotted using Cytoscape [90]. In order to access the connectivity of deregulated genes, the connectivity score was calculated as average absolute value of correlation coefficients of any gene pairs of the selected gene set. Permutation test was performed to evaluate the significance of the connectivity of deregulated genes. The connectivity score of the same number of randomly selected genes was calculated. Permutation was performed 1000 times, and p-value was obtained by counting the chance of obtaining equal or higher connectivity score in the permutation.

### Availability of supporting data

RNA-seq data sets in this study were uploaded to GEO with a series accession number GSE52652.

## Electronic supplementary material

Additional file 1: Figure S1: Quality control for the RNA-seq experiment. (A) Average percentage of reads mapping to unique or multiple locations in the genome. (B) Average percentage of reads with perfect match, or 1–2 bps mismatch to the genome. (C) Distribution and average standard deviation of read counts of genes. (D) Distribution and average standard deviation of reads per kilobase per million reads (RPKM) of genes, with lowly expressed genes removed. (PDF 357 KB)

Additional file 2: Table S1: List of differentially expression genes between *rx3* ^*-/-*^ and wild-type phenotype siblings. (XLSX 18 KB)
