# Supplementary material for: Genes and signaling networks regulated during zebrafish optic vesicle morphogenesis
Source: BMC Genomics. 2014 Sep 30;15(1):825. doi: 10.1186/1471-2164-15-825 (PMC4190348; doi:10.1186/1471-2164-15-825)
Supplement: Supplementary file 1 — Additional file 1: Figure S1: Quality control for the RNA-seq experiment. (A) Average percentage of reads mapping to unique or multiple locations in the genome. (B) Average percentage of reads with perfect match, or 1–2 bps mismatch to the genome. (C) Distribution and average standard deviation of read counts of genes. (D) Distribution and average standard deviation of reads per kilobase per million reads (RPKM) of genes, with lowly expressed genes removed. (PDF 357 KB) [file 12864_2014_6503_MOESM1_ESM.pdf]

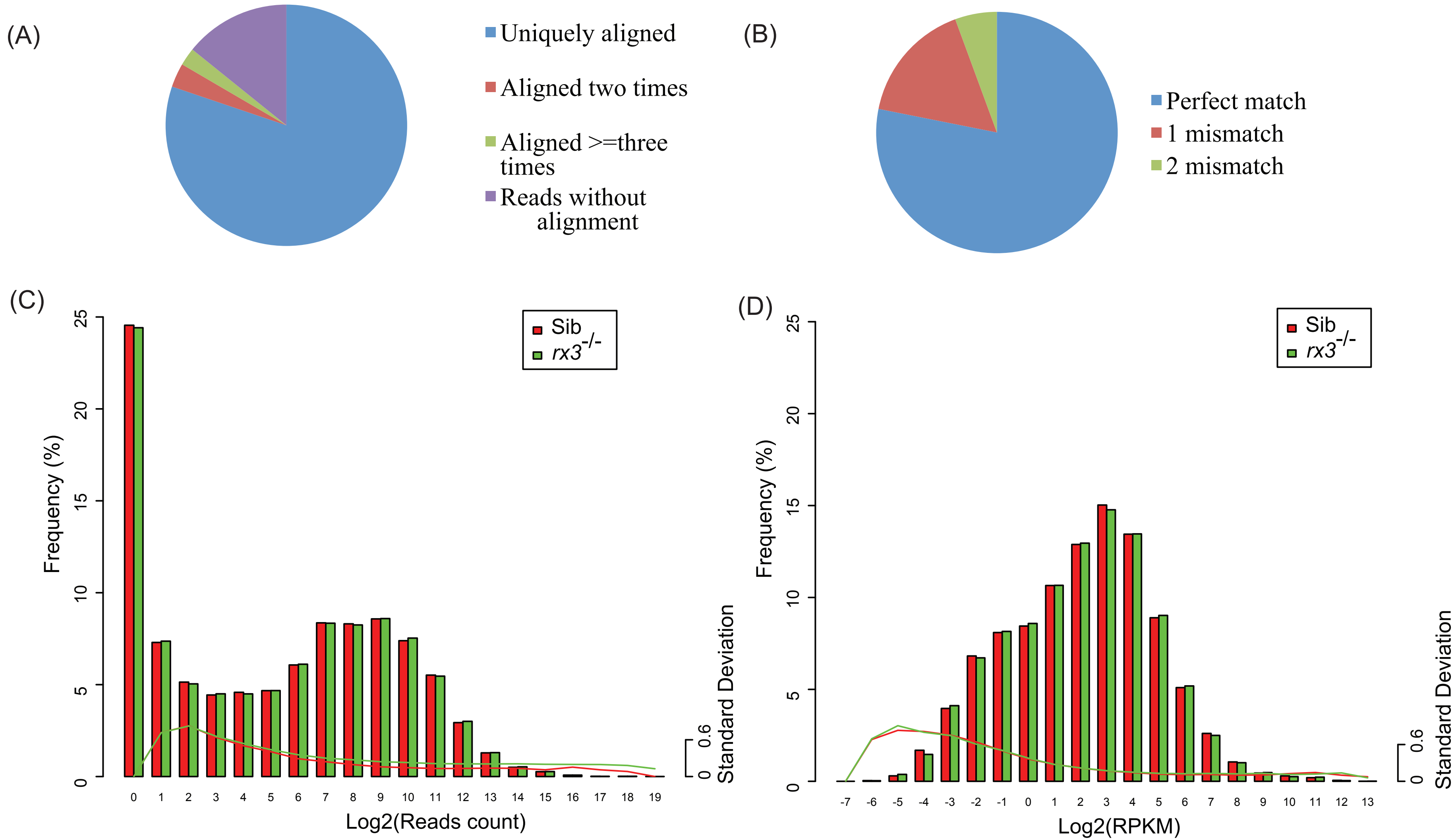
(A)
Uniquely aligned
Aligned two times
Aligned >=three times
Reads without alignment
(B)
Perfect match
1 mismatch
2 mismatch
(C)
Sib
rx3-/-
Frequency (%)
Standard Deviation
Log2(Reads count)
(D)
Sib
rx3-/-
Frequency (%)
Standard Deviation
Log2(RPKM)
